# A Strategy To Isolate Modifiers of *Caenorhabditis elegans* Lethal Mutations: Investigating the Endoderm Specifying Ability of the Intestinal Differentiation GATA Factor ELT-2

**DOI:** 10.1534/g3.118.200079

**Published:** 2018-03-28

**Authors:** Tobias Wiesenfahrt, Jingjie Duanmu, Frances Snider, Don Moerman, Vinci Au, Erica Li-Leger, Stephane Flibotte, Dylan M. Parker, Craig J. Marshall, Erin Osborne Nishimura, Paul E. Mains, James D. McGhee

**Affiliations:** * Department of Biochemistry and Molecular Biology, Alberta Children’s Hospital Research Institute, Cumming School of Medicine, University of Calgary, Calgary, Alberta, Canada T2N 4N1; † Department of Zoology and Michael Smith Laboratories, University of British Columbia, Vancouver, British Columbia, Canada V6T 1Z4; ‡ Department of Biochemistry and Molecular Biology, Colorado State University, Fort Collins, Colorado 80523

**Keywords:** *C. elegans*, intestine, endoderm specification, ELT-2 GATA factor, *tasp-1*, taspase, *pqn-82*, Mutant Screen Report

## Abstract

The ELT-2 GATA factor normally functions in differentiation of the *C. elegans* endoderm, downstream of endoderm specification. We have previously shown that, if ELT-2 is expressed sufficiently early, it is also able to specify the endoderm and to replace all other members of the core GATA-factor transcriptional cascade (END-1, END-3, ELT-7). However, such rescue requires multiple copies (and presumably overexpression) of the *end-1p*::*elt-2* cDNA transgene; a single copy of the transgene does not rescue. We have made this observation the basis of a genetic screen to search for genetic modifiers that allow a single copy of the *end-1p*::*elt-2* cDNA transgene to rescue the lethality of the *end-1end-3* double mutant. We performed this screen on a strain that has a single copy insertion of the transgene in an *end-1end-3* background. These animals are kept alive by virtue of an extrachromosomal array containing multiple copies of the rescuing transgene; the extrachromosomal array also contains a toxin under heat shock control to counterselect for mutagenized survivors that have been able to lose the rescuing array. A screen of ∼14,000 mutagenized haploid genomes produced 17 independent surviving strains. Whole genome sequencing was performed to identify genes that incurred independent mutations in more than one surviving strain. The *C. elegans* gene *tasp-1* was mutated in four independent strains. *tasp-1* encodes the *C. elegans* homolog of Taspase, a threonine-aspartic acid protease that has been found, in both mammals and insects, to cleave several proteins involved in transcription, in particular MLL1/trithorax and TFIIA. A second gene, *pqn-82*, was mutated in two independent strains and encodes a glutamine-asparagine rich protein. *tasp-1* and *pqn-82* were verified as loss-of-function modifiers of the *end-1p*::*elt-2* transgene by RNAi and by CRISPR/Cas9-induced mutations. In both cases, gene loss leads to modest increases in the level of ELT-2 protein in the early endoderm although ELT-2 levels do not strictly correlate with rescue. We suggest that *tasp-1* and *pqn-82* represent a class of genes acting in the early embryo to modulate levels of critical transcription factors or to modulate the responsiveness of critical target genes. The screen’s design, rescuing lethality with an extrachromosomal transgene followed by counterselection, has a background survival rate of <10^−4^ without mutagenesis and should be readily adapted to the general problem of identifying suppressors of *C. elegans* lethal mutations.

The classical Waddington landscape (Figure 1) provides a metaphor for the decisions made by a developing embryo. Waddington envisioned that the precise shape of this landscape reflected genes acting “beneath the surface”, determining whether the walls of the different valleys or cell trajectories are steep (stable robust pathways) or shallow (those more easily perturbed) (see Figure 5, Chapter 2 of Waddington 1957). For the well-defined case of the transcription factor cascade driving development of the *C. elegans* endoderm, we wish to identify background functions that contribute to the overall behavior and robustness of the regulatory network, thereby preventing an intestinal precursor from adopting incorrect cell fates because of random noise or environmental fluctuations. These background functions ensure that each transcription factor appears at the correct time, at the correct level, and with the appropriate interacting partners, thereby—to continue the metaphor—producing a “deep valley” in the Waddington landscape. The present study will describe a genetic screen aimed at identifying such background or secondary factors.

**Figure 1:**
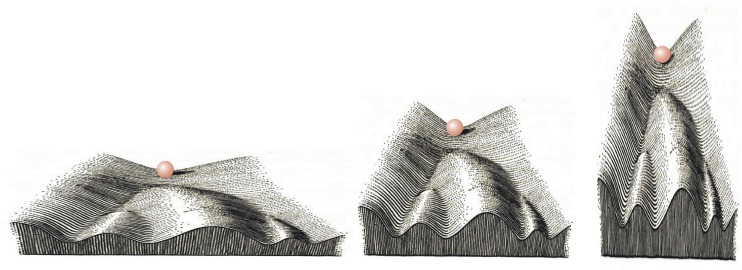
The Waddington developmental landscape (adapted from Figure 4, Chapter 2 of (Waddington 1957)) provides a metaphor for the developmental decisions made during embryonic development. Waddington envisaged the shape of the landscape to be determined by embryonic genes that could, in principle, change the contours of the landscape, such that valleys/trajectories could be shallow and sensitive to perturbation (left) or steep and more robust (right).

The *C. elegans* endoderm (intestine or E-lineage) forms as a simple clonal lineage under control of a cascade of GATA-type transcription factors (Figure 2A) (reviewed in (McGhee 2007; Maduro 2010; McGhee 2013; Maduro 2015, 2017)). The endoderm is normally specified by the redundant action of the GATA transcription factors END-1 and END-3, acting within the single E blastomere of the eight cell embryo (Zhu *et al.* 1997; Maduro *et al.* 2005; Maduro *et al.* 2007; Owraghi *et al.* 2009; Boeck *et al.* 2011; Maduro *et al.* 2015). Expression of *end-1* and *end-3* is controlled by a spatially patterned set of transcription factors, including the maternal SKN-1 (aided by its transient zygotic targets MED-1/2) (Bowerman *et al.* 1993; Maduro *et al.* 2015) and maternal POP-1 (whose levels and interacting co-factors are determined by intercellular signaling within the four-cell embryo) (Goldstein 1992, 1993; Lin *et al.* 1998; Maduro *et al.* 2005; Shetty *et al.* 2005). When there are two cells in the E lineage (2E cell stage; Figure 2A), END-1 and END-3 activate the gene encoding the GATA factor ELT-7 (Sommermann *et al.* 2010; Dineen *et al.* 2018). END-1, END-3 and ELT-7 then combine to activate the gene encoding the GATA factor ELT-2 at the early 4E cell stage (Wiesenfahrt *et al.* 2016; Dineen *et al.* 2018). Expression of *end-1* and *end-3* is transient and ceases by the 4-8E cell stage (Raj *et al.* 2010).

**Figure 2:**
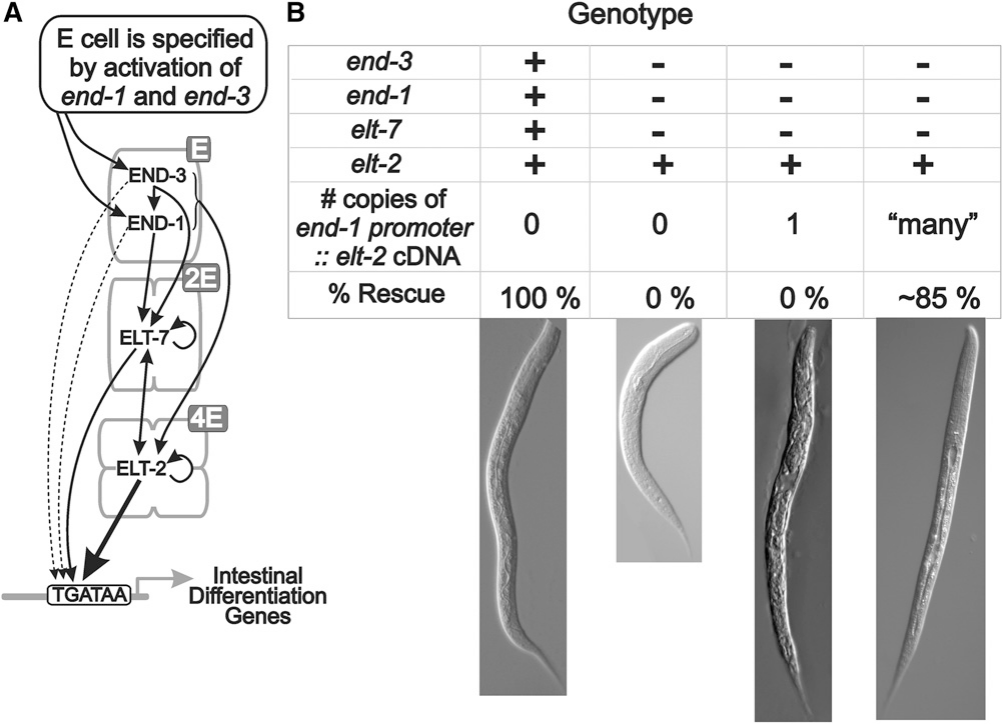
A. The zygotic cascade of GATA-type transcription factors that first specify (END-1 and END-3) and then differentiate (ELT-7 and ELT-2) the *C. elegans* endoderm lineage. Redrawn from Wiesenfahrt *et al.* (2016). B. Results supporting the rationale for the present screen (Wiesenfahrt *et al.* 2016). 100% of wildtype animals (*elt-7(+) end-1(+) end-3(+)*; *elt-2(+)*) survive. None of *elt-7(-) end-1(-) end-3(-)*; *elt-2(+)* survive (see also Owraghi *et al.* (2009)) unless they are transgenic for multiple copies of an *end-1p*::*elt-2* cDNA transgene. They do not survive if the transgene is present as a single copy.

ELT-2 normally controls genes associated with intestinal structure and function, *e.g.*, genes encoding digestive enzymes (Fukushige *et al.* 1998; McGhee *et al.* 2007; McGhee *et al.* 2009; Wiesenfahrt *et al.* 2016; Dineen *et al.* 2018). ELT-2 is not normally involved with the earlier events of endoderm specification. However, when ELT-2 is expressed earlier than normal (*i.e.*, under control of a transgenic *end-1* promoter introduced into an *elt-2(+)* background), ELT-2 is able to replace all other members of the core endoderm transcription factor hierarchy (*i.e.*, END-1, END-3 and ELT-7) to specify the *C. elegans* endoderm as well as drive intestinal differentiation (Wiesenfahrt *et al.* 2016). This ELT-2-only rescue is successful if the *end-1p*::*elt-2* cDNA construct is present in multiple copies (either as an integrated-chromosomal or a non-integrated-extrachromosomal transgenic array; Figure 2B). In contrast, a similar *end-1p*::*elt-2* cDNA construct does not rescue if it is present as a single copy, *i.e.*, a MosSCI insertion into a site that has been shown to be a permissive environment for early transcription (Frøkjaer-Jensen *et al.* 2012; Maduro *et al.* 2015; Wiesenfahrt *et al.* 2016). We hypothesize that successful rescue requires higher levels of ELT-2 protein than are provided by a single copy transgene. We further hypothesize that mutation in some “modifier” gene might increase levels of ELT-2 and/or increase the sensitivity of key ELT-2 target genes, thereby allowing the survival of an *end-1end-3* double mutant animal harboring the single copy *end-1p*::*elt-2* transgene. As will be described in this paper, a genetic screen was indeed able to identify genes that, when mutated, increase survival of *end-1end-3* embryos carrying the single copy *end-1p*::*elt-2* transgene and that could be viewed as altering the topography of the Waddington landscape. Two of these genes were identified by whole genome sequencing, without prior out-crossing, mapping or complementation testing. Mutations in both genes were indeed found to modestly increase levels of ELT-2 in the early embryo but increased ELT-2 levels may not be sufficient for rescue. We describe several straightforward ways in which the screen could be optimized to more fully map the developmental landscape of the *C. elegans* endoderm. The approach of rescuing a lethal mutation with an extrachromosomal transgene, followed by mutagenesis and counterselection against the extrachromosomal array, appears to be generally suited to identifying modifiers of lethal mutations in *C. elegans*. Fay and co-workers (Joseph *et al.* 2017) have recently described a conceptually similar screen to identify suppressors of a synthetic lethal moulting defect.

## Materials and Methods

### Strain maintenance, production and manipulation

Genotypes of the *C. elegans* strains used in this study are listed in Table 1. All strains were propagated on OP50-seeded NGM plates by standard methods (Brenner 1974). We note that several strains (*e.g.*, JM246, JM274) were deleted for the *elt-4* gene (allele *ca16*); loss of *elt-4* causes no detectable phenotype (Fukushige *et al.* 2003) and, for the purposes of the current study, we made no distinction between strains that were *elt-4(+)* or *elt-4(-)*. The *end-1end-3* rescued strains in our previous study were null for *elt-7* (Wiesenfahrt *et al.* 2016) but strain JM246 on which the current genetic screen was performed is *elt-7(+)*. Based on the known properties of ELT-7 (Sommermann *et al.* 2010; Riddle *et al.* 2013; Riddle *et al.* 2016; Dineen *et al.* 2018), we surmised that enhanced ELT-7 expression could provide a potential class of modifier mutations. In the event, no candidate *elt-7* mutations were detected; we also tested that all other candidates remained viable following *elt-7* RNAi; (a strain that depended on ELT-7 function provided a positive control that the *elt-7* RNAi had worked as expected).

**Table 1:** Genotypes of strains used

Strain	Genotype	Comments
JM229	*caIs85 I*; *elt-7(tm840) end-1(ok558) end-3(ok1448) V*; *elt-4(ca16) X*	*caIs85 I* is an integrated multicopy array containing [*end-1p*::*elt-2* cDNA (pJM513) + *rol-6* (pRF4) + *elt-2p*::GFP (pJM370)] and was previously used in (Wiesenfahrt *et al.* 2016).
JM246	*caSi2 II*; *elt-7(+) end-1(ok558) end-3(ok1448) V*; *elt-4(ca16) X*; *caEx10*	*caSi2 II* is a single copy of an *end-1p*::*elt-2* cDNA construct inserted into the *ttTi5605 II* site as described by Frøkjaer-Jensen *et al.* (2012). *caEx10* is a non-integrated extrachromosomal multicopy array containing [*end-1p*::*elt-2* cDNA (pJM513) + *myo-3p*::tdTomato (pJM464) + *heatshockpromoter*::*peel-1* (pMA122)] . JM246 is the starting strain for the current genetic screen.
JM247	*caSi2 II*	*caSi2 II* is a single copy insertion of an *end-1p*::*elt-2* cDNA construct into the *ttTi5605 II* site as described by Frøkjaer-Jensen *et al.* (2012).
JM274	*caSi2 II*; *tasp-1(ca18) III*; *end-1(ok558) end-3(ok1448) V*; *elt-4(ca16) X*; *caEx10*	
JM279	*caSi5 I*; *caSi2 II*; *caSi6 III*; *end-1(ok558) end-3(ok1448) V*; *caEx6*	Three separate single copy insertions of the *end-1p*::*elt-2* transgene; insertion sites are *oxTi185 I*, *ttTi5605 II*, and *oxTi444 III *(Frøkjaer-Jensen *et al. 2012*). *caEx6* is a non-integrated extrachromosomal multicopy array containing [*end-1p*::*elt-2*cDNA (pJM513) + *rol-6*(pRF4) + *elt-2p*::*GFP*(pJM370)]
JM280	*caSi2 II*; *pqn-82(gk3768) III*; *end-1(ok558) end-3(ok1448) V*; *elt-4(ca16) X*	
JM281	*tasp-1(ca18) III*	CRISPR/Cas9 induced 29 bp deletion in the *tasp-1* gene, as diagrammed on Figure 5A
JM282	*pqn-82(gk3768) tasp-1(ca18) III*	
JM283	*caSi5 I*; *caSi2 II*; *caSi6 III;*	Three separate single copy insertions of the *end-1p*::*elt-2* transgene; insertion sites are *oxTi185 I*, *ttTi5605 II*, and *oxTi444 III *(Frøkjaer-Jensen *et al. 2012**)*.
VC3801	*pqn-82(gk3768) III*	CRISPR/Cas9 disruption of the *pqn-82* gene

**Figure 3:**
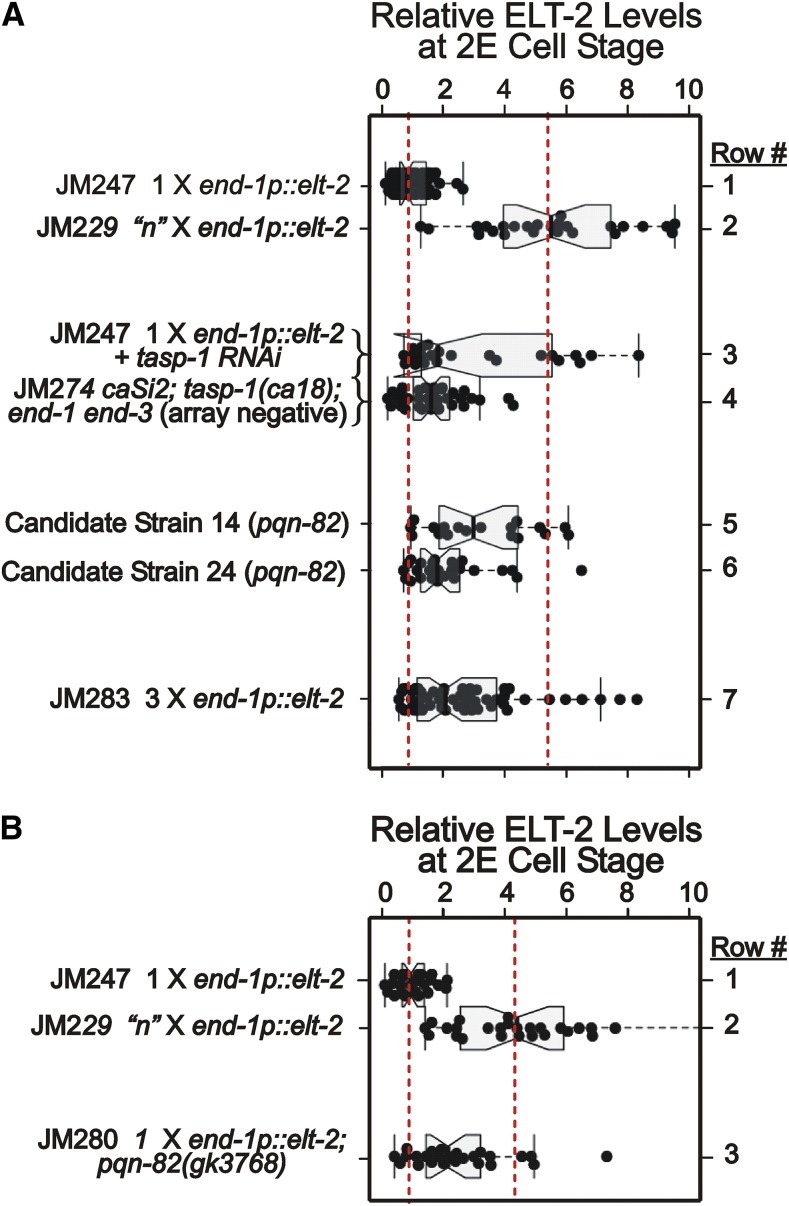
Levels of immunologically detectable ELT-2 protein measured at the 2E cell stage in embryos containing either single or multiple copies of an *end-1p*::*elt-2* transgene, with or without candidate mutations or RNAi. Each black circle corresponds to an individual embryo; the whiskers encompass all data points not judged to be outliers; the box represents the interquartile range (*i.e.*, 25 to 75% of the data); the notch represents ∼1.6(interquartile range)/(number of observations)^1/2^, such that the medians of two data sets are judged to be significantly different from each other if the corresponding notches do not overlap (McGill *et al.* 1978). Figures 3A and 3B represent independent experiments. Fluorescent intensities are normalized to the average intensity measured with JM247 (single copy of the *end-1p*::*elt*-2 transgene) in each experiment. Dashed red lines correspond to mean (normalized) ELT-2 levels measured for strain JM247 (single copy *end-1p*::*elt-2*) or strain JM229 (multiple integrated copies of *end-1p*::*elt-2*).

Multicopy extrachromosomal transgenic arrays were produced by standard gonadal injection (Mello *et al.* 1991). In particular, the extrachromosomal array (*caEx10*) that provides the basis of the current genetic screen was created by co-injecting three plasmids: pJM513 at 10 µg/ml (2162 bp of the *end-1* promoter fused to *elt-2* cDNA + 3′-UTR); pMA122 at 10 µg/ml (*peel-1* toxin under control of the heat shock promoter (Frøkjaer-Jensen *et al.* 2012)), and; pJM473 at 80 µg/ml (2.3 kb of the *myo-3* promoter fused to tdTomato). The single copy insert of the *end-1p*::*elt-2* cDNA (*caSi2* II (Wiesenfahrt *et al.* 2016)) was produced by MosSCI as described by Frøkjaer-Jensen *et al.* (2012); the transforming plasmid was pJM661 (1996 bp of the *end-1* promoter fused to *elt-2* cDNA + 3′-UTR inserted into pCFJ350) injected into strain EG6699 (Frøkjaer-Jensen *et al.* 2012). Single copy MosSCI inserts were also produced on Chromosome I (allele *caSi5* inserted into the *oxTi185* locus in strain EG8078) and Chromosome III (allele *caSi6* inserted into the *oxTi444* locus in strain EG8080). These three single copy insertions were combined by standard genetic crosses, monitoring the presence of the different alleles by PCR; strains were not subsequently checked for the presence of *unc-119*. Expression from the triple MosSCI strain appeared unstable and was eventually extinguished; measurements of ELT-2 levels shown in Figure 3A, row 7, as well as rescuing ability, were made immediately after the strain was produced.

RNAi by feeding was performed essentially as described by (Kamath and Ahringer 2003). Birefringent gut granules were scored using standard polarization optics. Survival rates (at 20°) for the various strains were measured as follows. Individual L4 larvae were placed on separate plates and transferred to a new plate every day for four days. For each plate from each original animal, total progeny were counted immediately after the adult was transferred to a new plate, dead/unhatched embryos were counted one day later and surviving adults were counted four days later. Dead/arrested larvae were estimated by subtracting the number of dead embryos and surviving adults from the number of total progeny. Overall survival rates were calculated by combining data from 3-5 original L4 larvae

### Mutagenesis and screening

We performed three separate mutagenesis protocols on L4/young adults of strain JM246: 1) 50 P0 animals were treated with 50 mM EMS; 2) 50 P0 animals were treated with a combination of 50 mM ENU and 25 mM EMS, and; 3) ∼300 worms were treated with 25 mM EMS. All treatments were performed in M9 buffer for 4h on a rotator at room temperature. Mutagenized animals were then washed three times with M9 by centrifugation and allowed to recover on an OP50-seeded NGM plate, either for three hours or overnight. Three to five mutagenized animals were then transferred to fresh OP50-seeded 100 mm plates and incubated at 20°; plates were cleared of food in ∼two weeks (3-5 generations), which should provide adequate time for a modifier mutation to become homozygous and the *hsp-peel-1* counterselection plasmid in the extrachromosomal array to be lost. Plates were then incubated at 34° for 2 hr to induce the PEEL-1 toxin as described in Frøkjaer-Jensen *et al.* (2012), killing essentially all worms that still contained the rescuing plasmid. Survivors were individualized the next day and progeny of individualized worms were screened for red fluorescence (indicating false positives) several days later. To estimate the total number of genomes screened, we measured the number of surviving fertile progeny produced by a 25 mM EMS-mutagenized JM246 adult to be roughly 15-20. (This low number reflects the loss of the rescuing array in ∼40% of progeny together with poor health of JM246 following mutagenesis, possibly worsened by the presence of the heatshock::*peel-1* construct.)

### Immunohistochemistry

ELT-2 protein levels in embryos were measured as described previously (Van Fürden *et al.* 2004; Wiesenfahrt *et al.* 2016), using the anti-ELT-2 monoclonal antibody 455-2A4. Images were recorded with a Hamamatsu Orca ER camera attached to a Zeiss Axioplan 2i microscope (40X objective); images for comparing different strains were collected with constant microscope/camera parameters. Total fluorescent intensity in the intestinal primordium (2E or 8E cell stage embryo) was measured (in arbitrary units) using ImageJ and corrected for background intensity measured elsewhere in the embryo. The probability that ELT-2 levels measured in one set of embryos have the same distribution of intensities as measured in JM247 embryos (single copy *end-1p*::*elt-2* transgene) was calculated by the Wilcoxon-Mann-Whitney rank test (Marx *et al.* 2016) implemented online (https://ccb-compute2.cs.uni-saarland.de/wtest/).

### Single molecule fluorescent *in situ* hybridization (smFISH)

Custom Stellaris FISH Probes were designed against *end-1* (oEO221) and *set-3* (oDMP123) mRNA using the Stellaris FISH Probe Designer (Biosearch Technologies, Inc., Petaluma, CA) available online at www.biosearch.com/stellarisdesigner. Probes were labeled with CalFluor610 and Quasar670 fluorophores. Probe sequences are listed in Table S2.

N2 and *pqn-82(−/−)* worms were grown at 20° to gravidity on NGM plates, bleached for embryos, re-suspended in -20° methanol, freeze cracked in liquid nitrogen, and fixed at -20° for 24-48 hr. A protocol was performed that combined (Ji and van Oudenaarden 2012; Shaffer *et al.* 2013) and Stellaris RNA FISH protocol for *C. elegans* (available online at www.biosearchtech.com/stellarisprotocols). Briefly, embryos were equilibrated in Stellaris Wash Buffer A followed by hybridization in Stellaris Hybridization Buffer containing 50 pmoles of each primer set (37°, overnight). Hybridization was followed by a wash in Wash Buffer A (37°, 30 min), DAPI staining in Wash Buffer A (37°, 30 min), a wash in Stellaris Wash Buffer B (room temp, 5 min), and final resuspension in N-propyl gallate mounting media. Embryos were mounted as described in (Ji and van Oudenaarden 2012) using either N-propyl gallate or VectaShield Diamond anti-fade to prevent photobleaching.

All smFISH images were acquired using a Cool Snap HQ2 camera on a DeltaVision Elite System (a modified, inverted IX71 Olympus microscope), with a 60x objective (NA 1.42) and SoftWorx software (Applied Precision) using fixed exposure and acquisition conditions and z-stacks at 0.25 µm thickness. Images were deconvoluted (Applied Precision). Quantitation was performed on stacks using FISH-Quant (Mueller *et al.* 2013). The ratio of *end-1* mRNA molecules per *set-3* mRNA molecules was calculated and the significance of differences between N2 and *pqn-82* embryos was assessed using the Student’s two-tailed *t*-test.

### CRISPR/Cas9 mutagenesis

A deletion in the K01G5.9/*tasp-1* gene was performed by co-CRISPR, using the *dpy-10* marker as described by (Arribere *et al.* 2014). Guide RNA (CCTTGCACATAGCAATTGG) was selected using the website: http://genome.sfu.ca/crispr/. Dpy and Rol worms were individualized and analyzed by PCR for deletions using primers flanking the guide RNA target site (Primers: oJM814: CCTTGCACATAGCAATTGG, oJM815: CCTTGCACATAGCAATTGG). The deletion of the *pqn-82* gene was performed by the *C. elegans* Gene Knock Out Lab using the method of Norris *et al.* (2015) to target Chromosome III: 1850135 to 1851020, using the following sequences flanking the deletion:

Upstream: TATCTCTTTTCTGGGCAAAGTTACAGAAGTTTTAAGAAAACTGGGTCAAG
Downstream: AGGAGCAATTGGCTCAGCAGCATCACCAGCACCAGCAGCAGCATGCTCAA

### Whole genome sequencing

Candidate populations (together with a population of the unmutagenized starting strain JM246) were grown from single animals. Genomic DNA was prepared using a standard protocol and resuspended in distilled water. Sequencing libraries were constructed using the Illumina NexteraXT library preparation kit and sequenced on either an Illumina HiSeq 2 × 100 Rapid Run or MiSequation 2 × 150 run. Paired sequence reads were mapped to the *C. elegans* reference genome version WS230 (www.wormbase.org) using the short-read aligner BWA (Li and Durbin 2010). Single-nucleotide variants (SNVs) were identified and filtered with the help of the SAMtools toolbox (Li *et al.* 2009). Variant calls also present in the parental strain were eliminated, each SNV was annotated with a custom Perl script, and gene information downloaded from WormBase version WS230.

### Data availability

The raw sequence data from this study have been submitted to the NCBI BioProject (http://www.ncbi.nlm.nih.gov/bioproject) under accession number PRJNA433018 and can be accessed from the Sequence Read Archive (SRA; https://www.ncbi.nlm.nih.gov/sra) with accession number SRP132234. Table S1 (summary of coding sequence variants) and Table S2 (probe sequences and raw counts for smFISH) are available at Figshare: https://doi.org/10.25387/g3.5972902.

## Results and Discussion

### Rationale for the modifier screen

We previously found that multiple copies of an *end-1p*::*elt-2* cDNA construct are able to rescue the lethality of an *end-1end-3* double mutant, whereas a single copy of a similar construct is unable to rescue (Figure 2B) (Wiesenfahrt *et al.* 2016). A possible cause of this differential rescue is overexpression of ELT-2 from the multicopy transgene in the early endoderm lineage. We therefore measured ELT-2 levels at the 2E cell stage using an anti-ELT-2 monoclonal antibody. ELT-2 protein can be detected in ∼1% of wildtype embryos at the late 2E cell stage and in 100% of wildtype embryos at the early 4E cell stage (Fukushige *et al.* 1998; Raj *et al.* 2010; Wiesenfahrt *et al.* 2016). In contrast, ELT-2 protein can be detected at the 2E cell stage (when the *end* genes are normally expressed) in 100% of embryos from either strain JM247 (single *caSi2* copy MosSCI insertion of the *end-1p*::*elt-2* cDNA transgene) or from strain JM229 (the integrated multiple copy array caIs85 of a similar *end-1p*::*elt-2* cDNA transgene) (Figure 3A rows 1 and 2, respectively). (Genotypes of key strains are summarized in Table 1.) As we suspected, ELT-2 levels at the 2E cell stage produced by the multicopy array are, on average, ∼six fold (5.5 +/− SD = 3.8) higher than levels produced by the single copy MosSCI insertion (p∼10^−16^). The large variability in fluorescence intensity is commonly seen when immunostaining *C. elegans* embryos by standard procedures, either because of variable permeablization of the eggshell, inability to select for a precise time point within the cell cycle, or intrinsic variability in gene expression. Nonetheless, we are confident of at least semi-quantitative interpretations. To support this claim, Figure 3B (rows 1 and 2) shows the results of measurements on the same two strains performed one year later than the measurements of Figure 3A; the ratio of ELT-2 levels was estimated at 4.7+/− SD = 3.6; (*P* < 10^−11^).

We designed a genetic screen to identify mutations that would allow *end-1end-3* mutant animals to survive with a single copy of the *end-1p*::*elt-2* cDNA construct. We refer to these putative mutations as “modifiers” rather than enhancers or suppressors, in order to avoid presumptions about mechanisms. The starting strain for the screen, JM246, is *caSi2 II*; *end-1end-3 V*; *caEx10*. Because the *caSi2* single copy *end-1p*::*elt-2* cDNA construct is insufficient to rescue *end-1end-3* lethality (Figure 2B), strain JM246 survives by virtue of the extrachromosomal multicopy transgenic array *caEx10*, which is derived from three plasmids: (i) the *end-1p*::*elt-2* cDNA construct able to rescue the *end-1end-3* lethality as multiple copies, and for counterselection; (ii) a tdTomato fluorescent reporter expressed under control of the body wall myosin *myo-3* promoter, and; (iii) a heatshock::*peel-1* construct. As described by Frøkjaer-Jensen *et al.* (2012), animals that harbor the heatshock::*peel-1* toxin construct can be efficiently killed by brief heat shock; (see also (Seidel *et al.* 2011; Joseph *et al.* 2017)). (In principle, a normal genomic copy of the *end-1* or *end-3* genes could have been used to rescue *end-1end-3* lethality in the starting strain; however, we chose to use an *end-1p*::*elt-2* construct in JM246 to avoid recombination from the multicopy array back into the genome, which would thereby produce false positives; this choice will be discussed in a later section.) We did not observe animals segregating from unmutagenized JM246 that did not also contain the extrachromosomal transgenic array (*i.e.*, < 1 in ∼10^4^ progeny are Non-Red and Non-Sensitive to heatshock). We note that ∼40% of JM246 progeny lose the rescuing extrachromosomal transgenic array in each generation; however, ∼45% of these array-negative (and non-propagating) embryos still express birefringent gut granules, the standard marker of endoderm specification and intestinal differentiation. In other words, the single copy *caSi2* insert must be providing some partial level of endoderm specification/differentiation functions and the strain seems optimally poised to detect modifiers. For comparison, the *end-1end-3* double mutant (in the absence of the *caSi2 end-1p*::*elt-2* transgene) shows 0% gut granule expression (Owraghi *et al.* 2009).

**Figure 4:**
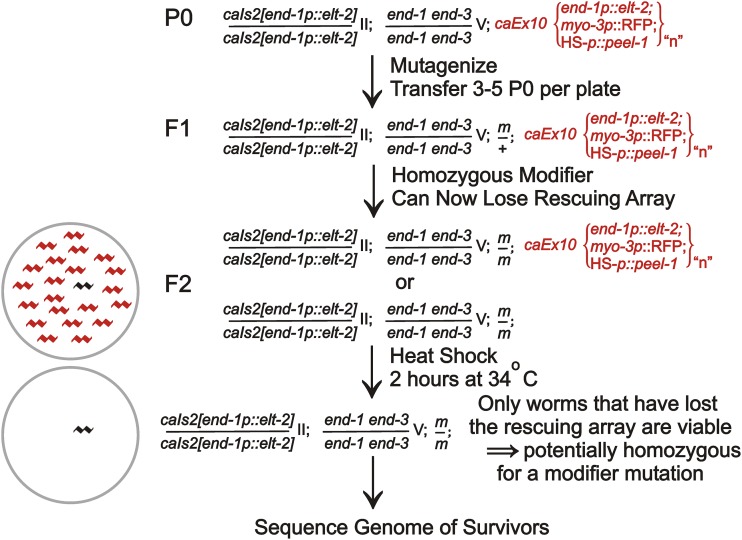
Outline of the genetic screen to identify modifiers of the single copy *end-1p*::*elt-2* cDNA transgene. A more detailed description of the rationale of each step is provided in the main text.

The overall plan of the screen is diagrammed in Figure 4. Young adults from strain JM246 (*caSi2 II*; *end-1end-3 V*; *caEx10*) were exposed to one of three mutagenic regimes (see below), placed 3-5 individuals per 100 mm plate and incubated at 20° until the bacterial food was exhausted. This took 10-14 days or an estimated 3-4 generations, which should allow either maternal or zygotic modifier mutations to become homozygous, together with or followed by random loss of the rescuing array. Plates were then heatshocked (34° for 2 hr), thereby killing all animals that had not lost the rescuing transgenic array. Array loss was verified by confirming that survivors were Non-Red. Rescued strains were established from a single survivor per plate to assure independence.

We performed three separate rounds of mutagenesis on young adults of strain JM246 (*caSi2 II*; *end-1end-3 V*; *caEx10*). Round 1 used 50 mM ethyl methane sulfonate (EMS) to mutagenize 50 P0 animals and produced 3 candidate strains; round 2 used 25 mM EMS + 25 mM ethylnitrosourea on 50 animals and produced 1 candidate strain; round 3 used 25 mM EMS on 300 adult animals and produced 13 candidate strains. We estimate that we screened 12,000-16,000 haploid genomes (see Methods). Brenner (1974) estimated a total forward mutation rate of 5 × 10^−4^ per gene when adults were exposed to 50 mM EMS. Estimating the mutation rate in the present screen to be approximately half of this (see Methods), we would expect that, if a particular gene could mutate to a simple loss of function allele that allowed survival, this gene would be detected 3-4 times,. As will be described below, two genes were mutated multiple times (two and four times), consistent with loss of function alleles. Modifiers in the remaining 11 candidate strains could thus represent less frequent change-of-function alleles.

### Whole genome sequencing identifies candidate modifier genes

We sequenced the full genomes of each of the 17 independent candidate strains, with the rationale that candidate modifiers could be identified if the same gene was mutated independently in more than one strain. Sequencing was performed at a median coverage of 35-fold (range = 25-to-58-fold); coding sequence variants are collected in Supplementary Material Table S1. Table 2 collects the identities of all genes that sustained two or more coding variant mutations. Table 2 also contains the calculated probability that each of the candidates was identified by chance, based on estimates of the intrinsic mutability of the identified genes from the Million Mutation Project (MMP) (Thompson *et al.* 2013). None of the strains included promoter or coding region mutations in the endogenous *elt-2* locus nor, as expected because the starting alleles are deletions, revertants of *end-1/3*. We chose two candidates, K01G5.9 (renamed *tasp-1* below) and *pqn-82*, to follow up in more detail. For the moment, we have not pursued other two-hit genes, *e.g.*, Y18H1A.8, because of lack of transcripts in the early embryo, lack of promising annotation, because several of these candidate strains were also mutated for *tasp-1* or *pqn-82*, and because of increasing probability that these genes were mutated by chance alone.

**Table 2:** Summary of candidate strains that have >1 mutation in any single gene. MMP denotes the Million Mutation Project (Thompson *et al.* 2013)

Gene	LG	Map	# in MMP	Rank in MMP	# Strains	Probability*^a^*
K01G5.9	III	5	6	12026	4	2.E-07
Y18H1A.8	I	−18.3	3	17836	2	3.E-04
*pqn-82*	III	−15.96	4	14376	2	5.E-04
*wrt-7*	V	7.82	6	10909	2	1.E-03
*clec-6*	III	−2.41	9	7605	2	3.E-03
B0462.1	V	16.07	12	5218	2	4.E-03
*col-180*	X	6.35	15	3130	2	7.E-03
*ptr-1*	V	1.91	18	2040	2	1.E-02
*ptr-19*	III	5.38	30	672	2	2.E-02
*panl-2*	III	−0.87	30	612	2	2.E-02
*odr-1*	X	12.72	33	613	2	3.E-02
*wnk-1*	IV	4.11	35	388	2	3.E-02
*rrf-3*	II	0.74	36	393	2	3.E-02
*pgp-9*	V	13.19	38	38	2	4.E-02
*gei-3*	X	8.48	38	312	2	4.E-02
*trr-1*	II	3.91	76	73	2	1.E-01
T06D8.1	II	3.36	82	32	2	1.E-01
*let-805*	III	−10.86	121	121	2	2.E-01
*dig-1*	III	−0.95	250	2	2	3.E-01

a Probability for each candidate gene of finding (# Strains) among 17 candidates. Calculated from the number of hits from the MMP. Since the current mutagenesis regime is weaker than that used in the MMP, the calculated probabilities shown in the table are over-estimated.

### Validation and initial characterization of two candidate modifier genes

#### K01G5.9/tasp-1:

**Figure 5:**
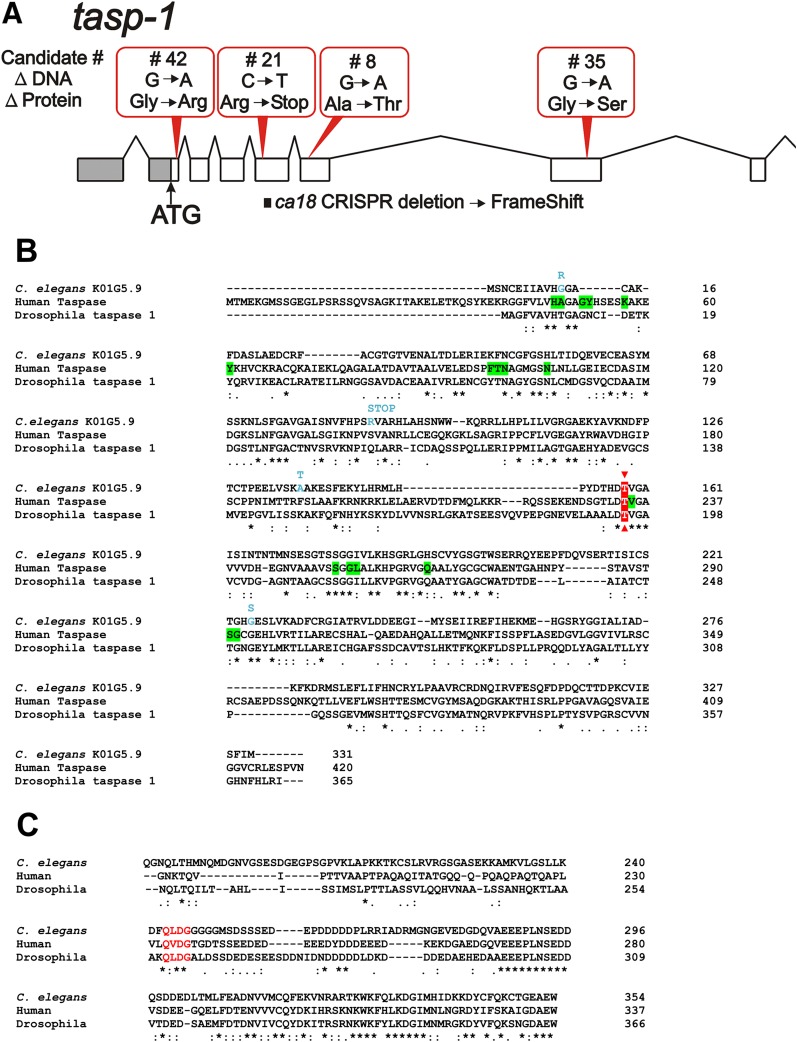
A. Four independent mutations identified in *tasp-1* are shown above the gene. The CRISPR mutation (*ca18*) introduced into the gene is also shown (below). B. Alignment (Clustal Omega performed at www.ebi.ac.uk with default settings) of the protein sequences from *C. elegans tasp-1*, human Taspase1 and Drosophila taspase 1. The Threonine residue highlighted in red depicts the active site nucleophile, which lies immediately downstream of the aspartic acid residue in the autocleavage site. Residues highlighted in green on the sequence of human Taspase1 have been identified as part of the active site and are not found in asparaginases (Khan *et al.* 2005). Changes in the amino acid sequence resulting from the four candidate mutations identified in the current screen are shown in blue. C. Alignment (Clustal Omega) of the C-terminal portions of TFIIA, showing that the taspase cleavage site known in humans and Drosophila is also conserved in *C. elegans* (highlighted).

This gene was independently mutated four times in our set of 17 candidate genomes and we estimate that the probability of this happening by chance is ∼2x10^−7^. The rate at which these mutations were found (∼1/3500 genomes screened) suggest that they represent loss of function alleles and this will be supported below. K01G5.9 is annotated in WormBase (WS261) as an L-asparaginase but we suggest that it is better described as the *C. elegans* homolog of Taspase; K01G5.9 has now been designated *C. elegans tasp-1*. Taspase is a conserved threonine-aspartic acid protease that cleaves mammalian and Drosophila MLL1/trithorax, as well as other nuclear proteins (*e.g.*, TFIIA) associated with chromatin and transcription; mutations in human Taspase1 have been associated with certain forms of cancer, particularly those of the head and neck (Hsieh *et al.* 2003a; Hsieh *et al.* 2003b; Khan *et al.* 2005; Takeda *et al.* 2006; Zhou *et al.* 2006; Oyama *et al.* 2013; Niizuma *et al.* 2015; Takeda *et al.* 2015; Stauber *et al.* 2016; Gribko *et al.* 2017). The four independent mutations are placed on the *tasp-1* genomic locus as shown in Figure 5A. Sequence alignments of taspases from *C. elegans*, humans and Drosophila are shown in Figure 5B; residues highlighted in green are part of the human taspase active site and distinguish taspases from asparaginases (Khan *et al.* 2005). Highlighted in red is the conserved threonine residue that becomes, following autoproteolysis, the N-terminal nucleophile that cleaves after the adjacent aspartate residue (Khan *et al.* 2005). Individual mutations in the four candidate strains are denoted in blue; consistent with loss of function alleles, one mutation produces a stop codon and two further mutations occur either in or adjacent to conserved residues implicated in catalysis. *tasp-1* expression appears wide-spread throughout the embryo and not obviously restricted to the developing endoderm. Levels of *tasp-1* transcripts are detectable in all life stages but appear to be highest in the early embryo (probably the oocyte) (Gerstein *et al.* 2010; Hashimshony *et al.* 2015; Cao *et al.* 2017).

As an initial validation of *tasp-1* as a candidate modifier, we performed feeding RNAi on the parent strain JM246 (single copy *end-1p*::*elt-2* rescued by the multicopy array). A low level of rescue was observed; 4% of animals that had lost the rescuing array survived to adulthood compared to 0% for the *ges-1* RNAi control. Furthermore, the average ELT-2 protein levels in 2E cell stage (array negative) embryos were increased ∼threefold (3.1 +/− SD = 3.0; *P* < 10^−5^) (Figure 3A, row 3). We then used CRISPR/Cas9 to produce a 29 bp deletion (allele *ca18)* in the endogenous *tasp-1* gene (Figure 5A; see Methods), which causes a frame shift mutation leading to a premature translational stop and likely represents a null. The *tasp-1(ca18)* allele was introduced into the single copy MosSCI *caSi*2 [*end-1p*::*elt-2*]; *end-1end-3* background to produce strain JM274. This strain shows 12% rescue and ∼twofold (1.8 +/− SD = 1.4; *P* < 10^−3^) increase in ELT-2 protein levels measured at the 2E cell stage (Figure 3A, row 4); although these protein levels fall within the range produced by the multicopy transgene (shown in Figure 3A, row 2), survival rates are much lower. Nonetheless, we conclude that loss of function in the *tasp-1* gene does indeed allow a single copy of the *end-1p*::*elt-2* cDNA transgene to rescue the *end-1end-3* lethality, at least partially.

Substrates and preferred cleavage sites of the taspase enzyme in *C. elegans* are not yet known. However, there are 239 proteins in WormBase (WS250) that contain the cleavage site (Q[F/I/L/M]D↓G) determined for Drosophila Taspase (Stauber *et al.* 2016)). For example, PQN-51, the *C. elegans* homolog of TFIIA (Blackwell and Walker 2006), contains an internal QMDG sequence that aligns with similar sequences in human and Drosophila TFIIA that are known to be cleaved by Taspase (Zhou *et al.* 2006) (Figure 5C). ELT-2 does not contain an obvious candidate cleavage site, suggesting that increased levels of ELT-2 in the early embryo do not result from abolishing direct TASP-1 cleavage of ELT-2. Similarly, candidate taspase cleavage sites could not be detected in other factors acting in the core endoderm specification pathway (SKN-1, POP-1, WRM-1, SYS-1, MED-1/2, END-1/3 and ELT-7).

We note an intriguing feature of all the *caSi2*; *tasp-1*; *end-1end-3* strains: they appear “mortal” and eventually die out after multiple (∼10) generations. This ultimate mortality was seen with all four of the original candidate strains, with the strain reconstituted from the CRISPR/Cas9 *ca18* deletion, as well as with array-negative segregants from JM246 produced and propagated by continued exposure to *tasp-1* RNAi. (Similar mortality was not obvious with *tasp-1(ca18)* animals with an otherwise wildtype background; however, an eventual but impenetrant mortality cannot be ruled out). Could *caSi2*; *tasp-1*; *end-1end-3* mortality possibly be associated with more general phenomena such as mortal germlines and “transgenerational epigenetic inheritance”? Work from several laboratories has shown that mutations in three genes, *hrde-1*, *met-2* and *prg-1*, are associated with both mortal germlines and transgenerational RNAi (reviewed in (Brown and Montgomery 2017)). Both HRDE-1 and MET-2 proteins contain sequence motifs that match the Q[F/I/L/M]D↓G consensus cleavage site for Drosphila taspase (QMDG and QFDG respectively); PRG-1 protein contains a QVDG sequence and we note that the second position V is part of the consensus cleavage site of human Taspase1. Given that ∼1% of *C. elegans* proteins have a candidate taspase cleavage motif, it seems improbable that three proteins chosen for an independent property all could be (potential) taspase targets.

#### pqn-82:

The second validated candidate was *pqn-82* (Y39A3CR.7), annotated in Wormbase as a “prion-like glutamine/asparagine rich domain bearing protein”. We estimate the probability that *pqn-82* was mutated twice by chance in our screen as 5x10^−4^. *pqn-82* transcripts are high in oocytes and early embryos, apparently in most cell lineages, but decline sharply by mid-embryogenesis; transcripts can be detected again when adults become gravid (Gerstein *et al.* 2010; Hashimshony *et al.* 2015; Cao *et al.* 2017).

There are no obvious *pqn-82* homologs outside of nematodes; even within nematodes, conservation is modest (Blast P scores >10^−7^). Nonetheless, other members of the PQN family have been associated with transcription and chromatin: for example, PQN-49/LET-19 is related to the conserved transcriptional coactivator subunit TRAP240, PQN-50/SEA-2 is a zinc finger protein involved in interpreting the X:A chromosomal ratio for sex determination and dosage compensation, PQN-81 has been redesignated *ssl-1* (Swi2/Snf2 like) and PQN-85 is homologous to Drosophila NIPPED-B involved in DNA repair and chromosome mechanics. PQN-51 is the *C. elegans* homolog of the general transcription factor TFIIA, noted above to be a candidate target of Taspase (Figure 5C).

**Figure 6:**
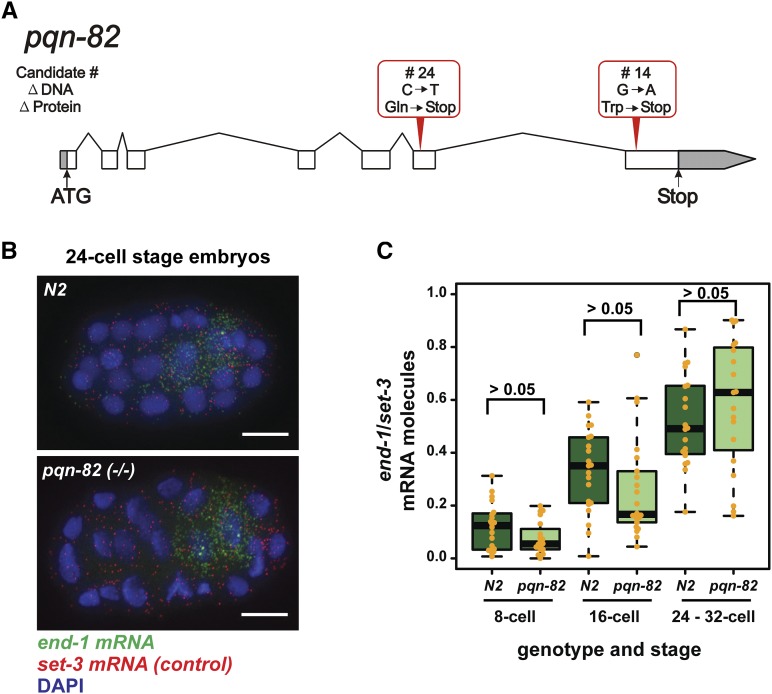
A. The two independent mutations identified in the structural gene of *pqn-82* are shown above the gene. The genomic coordinates for the altered base pairs are 1,851,047 for Candidate # 24 and 1,852,218 for Candidate # 14. B. *end-1* mRNA (green) in wild-type (N2) and *pqn-82* embryos as imaged by single molecule fluorescence *in situ* hybridization (smFISH). Ubiquitously expressed *set-3* mRNA transcripts (red) were co-hybridized as a control, and nuclei were stained with DAPI. Scale bar is 10 μm. C. Single molecules of *end-1* and *set-3* mRNA that were imaged by smFISH (B) were quantitated at three stages of embryonic development. Levels of *set-3* transcripts stayed consistent across stages (mean of 444 molecules/embryo) but levels of *end-1* transcripts increased (means of 47, 109, 214 molecules/embryo at 8-cell, 16-cell, and 32-cell stages). However, the relative amount of *end-1* mRNA in N2 and *pqn-82(−/−)* embryos was not significantly different for each stage-specific, pair-wise comparison as calculated by Student’s two-tailed *t*-test (20 embryos per stage and genotype). Upper boxplot whiskers represent the lesser of either the greatest value point or the upper quartile plus 1.5 times the interquartile range; lower whiskers represent the reverse. The data derive from one of two independent replicates, both of which reach the same conclusions.

As shown on Figure 6A, both *pqn-82* mutations isolated in the screen are nonsense alleles with premature termination toward the 3′-end of the gene and presumably cause a loss-of-function. The two candidate strains show 40–50% rescue and ELT-2 protein levels are increased two-to-three fold at the 2E cell stage (Figure 3A; 3.3 +/− SD = 2.5 for candidate strain 14 (row 5) and 2.2 +/− SD = 1.8 for candidate strain 24 (row 6); *P* < 10^−8^ and p∼10^−6^, respectively). RNAi performed against *pqn-82* on the parent strain JM246 does indeed lead to rescue, *i.e.*, production of *caSi2*; *end-1end-3* offspring that can now survive without the multicopy extrachromosomal transgenic array (data not shown). We disrupted the *pqn-82* gene (strain VC3801 *pqn-82(gk3768)*) using the CRISPR-Cas9 gene editing system as described by (Norris *et al.* 2015) (see Methods). *pqn-82(gk3768)* has no obvious phenotype on its own but the mutation is able to rescue *end-1end-3* lethality. That is, strain JM280 *caSi2 II*; *pqn-82(gk3768) III*; *end-1end-3 V* shows 50+/− SD = 8% rescue (brood size = 81+/−46, n = 5); we verified that this rescue requires the *caSi2* MosSCI insertion. As shown in Figure 3B row 3, ELT-2 protein levels at the 2E cell stage are increased 2.5 +/− SD = 2.2 fold (*P* < 10^−4^). Of the JM280 embryos that fail to hatch, 88% still express gut granules, further evidence for partial endoderm rescue; (recall that ∼43% of non-rescued embryos from the starting strain JM246 express gut granules). We saw no evidence for eventual mortality in the *caSi2*; *pqn-82*; *end-1end-3* strains, in contrast to corresponding strains with *tasp-1* mutations.

Two models by which *pqn-82* loss of function might rescue viability in animals with single-copy *end-1p*::*elt-2* transgenes are: (i) by stimulating higher expression of the *elt-2* coding region from the *end-1* promoter, or; (ii) by affecting the potency of the ELT-2 protein downstream of its transgenic expression. To differentiate between these scenarios, we tested whether *end-1* promoter activity was altered in *pqn-82* mutants. We did **not** observe higher levels of *end-1* mRNA transcripts in 8-cell, 16-cell, or 24–32 cell stage embryos as measured by single molecule fluorescent *in situ* hybridization (smFISH) (Figure 6B,C). This result suggests that *pqn-82* could function downstream of the *end-1p*::*elt-2* transgene, for example, on the endogenous *elt*-2 gene, on ELT-2 translation or stability, or on some other downstream target or set of targets.

### Do tasp-1 and pqn-82 function in parallel pathways?

We asked if *tasp-1* and *pqn-82* act in parallel pathways. If so, phenotypes of null alleles should show synergistic interactions. Table 3 collects brood sizes and survival rates of progeny produced by the strains that have lost the function of *tasp-1* and/or *pqn-82*. Both single mutants show lower brood sizes and a low degree of lethality (4–6%) compared to wildtype; both effects appear at most “additive” in the *pqn-82tasp-1* double mutant rather than synergistic. For strain JM280 *caSi2*; *pqn-82(gk3768)*; *end-1end-3*, roughly 50% of the laid embryos will reach adulthood. For strain JM274 *caSi2*; *tasp-1(ca18)*; *end-1end-3*, 19% of array-negative animals reach adulthood. When both *pqn-82* and *tasp-1* are removed, an intermediate number (30%) of array-negative animals reach adulthood. Considering the large inter-brood variability and the possibility that *tasp-1* RNAi might be less effective than the *tasp-1(ca18)* knockout, we suggest that the data are most consistent with a model in which *tasp-1* and *pqn-82* act in the same pathway with respect to brood size and viability.

**Table 3:** Brood size and progeny survival to adulthood (number and percentage) associated with strains that have lost *pqn-82* and/or *tasp-1*

Strain	Genotype / RNAi	Average Brood Size ± SD.	Progeny that Reach Adulthood		Broods Counted
			Average #	Average %	
N2	wildtype	287 ± 15	286 ± 15	99 ± 1	5
VC3801	*pqn-82(gk3768)*	226 ± 10	216 ± 14	96 ± 3	5
JM281	*tasp-1(ca18)*	173 ± 25	163 ± 34	94 ± 6	3
JM282	*pqn-82(gk3768) tasp-1(ca18)*	151 ± 20	138 ± 18	92 ± 4	4
JM280*^a^*	*caSi2*; *pqn-82(gk3768)*; *end-1 end-3*	163 ± 87	81 ± 46	50 ± 8	5
JM274*^a^*	*caSi2*; *tasp-1(ca18)*; *end-1 end-3*	104 ± 61	26 ± 22	19 ± 15	5
JM280	*caSi2*; *pqn-82(gk3768)*; *end-1 end-3*; *tasp-1 RNAi*	120 ± 46	30 ± 8	30 ± 15	5

a JM274 and JM280 are maintained by the presence of the extrachromosomal array *caEx10*, which rescues the *end-1 end-3* lethality. For these two strains, as well as for JM280 + *tasp-1 RNAi*, the progeny counted were only those that had lost the *caEx10* array (*i.e.*, that were NonRed).

We next tested whether *tasp-1* and *pqn-82* act in parallel pathways with respect to endoderm specification/differentiation. The maternally provided SKN-1 transcription factor specifies the fate of the EMS cell, the progenitor of the entire endoderm and of a fraction of pharynx and bodywall muscle (Bowerman *et al.* 1992; Bowerman *et al.* 1993; Raj *et al.* 2010). Removal of SKN-1, either by RNAi or by mutation, causes 100% embryonic lethality. However, birefringent gut granules, the standard marker for endoderm specification/differentiation, can still be detected in roughly one-third of these embryos, reflecting mostly the parallel POP-1 dependent pathway of endoderm specification (Maduro *et al.* 2005; Shetty *et al.* 2005). As shown in Table 4, the fraction of arrested embryos that show gut granules increases from 33% in *skn-1(RNAi)* to 63% in *skn-1(RNAi)*; *tasp-1(ca18)*. In contrast, deletion of *pqn-82* does not lead to enhanced expression of endoderm markers following *skn-1* RNAi (Table 4), consistent with *tasp-1* and *pqn-82* functioning in part in different pathways. We also note that the increased expression of gut granules in the *tasp-1(ca18) skn-1* RNAi embryos provides evidence that TASP-1 has functions on endoderm specification/differentiation in *C. elegans* beyond the transgenes on which the present screen is based.

**Table 4:** Influence of *tasp-1* and *pqn-82* mutations on the ability of *skn-1* RNAi embryos to produce gut granules. RNAi was administered by feeding the parent strains and inspecting arrested embryos by birefringence

Strain	gut granule **+**	gut granule **-**	n	% gut granule **+**
N2	181	365	546	33%
*tasp-1(ca18)*	513	308	821	63%
*pqn-82(gk3768)*	145	325	470	31%

### The rescuing ability of tasp-1 and pqn-82 is not solely due to increases in ELT-2 protein levels

Both *tasp-1* and *pqn-82* loss-of-function lead to an increase in the level of ELT-2 protein detectable at the 2E cell stage of the embryo (Figures 3A and B). Although ELT-2 levels in single copy *end-1p*::*elt-2* embryos were comparable for *tasp-1* and *pqn-82* mutants, rescue was considerably higher for *pqn*-82, suggesting that ELT-2 levels may not be the sole factor determining rescue. To test whether increased ELT-2 by itself is sufficient to cause rescue, we constructed strain JM279 carrying three separate copies, each on a different chromosome, of an *end-1p*::*elt-2* cDNA insert. Figure 3A, row 7, shows that the ELT-2 protein levels at the 2E cell stage are increased roughly threefold (3.2 +/− SD = 4; *P* < 10^−9^) relative to the single insertion used for the mutagenized parental strain. However, when these three copies were introduced into the *end-1end-3*; *caEx10* background and after counter-selection against the rescuing array, we were unable to detect rescued progeny, suggesting that ∼threefold increase in ELT-2 levels at the 2E cell stage is insufficient, by itself, to rescue *end-1end-3* lethality. The average level of ELT-2 protein at the 2E cell stage of these triple MosSCI embryos is roughly half of the average ELT-2 level detected in the same stage embryos of the multicopy strain JM229 (compare rows 7 and 3 of Figure 3A), suggesting there could be a threshhold level of ELT-2 needed to survive. However, the distributions of ELT-2 levels in individual embryos strongly overlap in the two strains and the upper range of the non-rescuing triple MosSCI strain is above that of the rescuing *pqn-82* strain (Figure 3A). We suggest that *tasp-1* and *pqn-82* may have an additional function(s) beyond simply increasing ELT-2 protein levels in the early embryo. Perhaps a time window, rather than a concentration threshold, could be critical and the *tasp-1* or *pqn-82* strains (as well as the original multicopy strain) could produce more ELT-2 earlier in the 2E cell cycle than the simple three-copy MosSCI insertions. We have previously commented that ELT-2 protein cannot be detected at the 1E cell stage in the JM229 multicopy strain (*i.e.*, at the stage when END-1/3 are normally first detected (Zhu *et al.* 1997; Raj *et al.* 2010)), even though the endoderm ultimately ends up being specified (Wiesenfahrt *et al.* 2016). Perhaps even more of a delay in the appearance time of ELT-2 is sufficient to abolish rescue.

### Estimating the background rate of “false positives” or “bypass mutations”

Before repeating the present screen on a scale that could approach saturation, we wished to assess whether identification of “false-positive” modifiers ever becomes a limitation. In other words, does the screen turn up large numbers of “bypass mutations” that do not depend on the behavior of the single copy *end-1p*::*elt-2* MosSCI insert on which the current screen was based? We repeated the basic screen described above but replaced the *caSi2* single copy MosSCI allele of *end-1p*::*elt-2* with an integrated multicopy array of a construct in which the *end-1* promoter now drives expression of a cDNA coding for the hypodermal GATA factor ELT-3 (Gilleard *et al.* 1999; Gilleard and McGhee 2001). Within the embryo, ELT-3 and ELT-2 clearly have distinct sets of transcriptional targets (Fukushige *et al.* 1998; Gilleard and McGhee 2001) and it would seem unlikely (but extremely interesting) to identify mutations that could “modify” normal ELT-3 hypodermal function such that it could now specify endoderm. Relevant to the current discussion, this screen would also produce *end-1/3* bypass mutations. As for the major screen, *end-1end-3* lethality was rescued in the parent by an extrachromosomal array containing *end-1p*::*elt-2*, *myo-3p*::*RFP* and *heatshock*::*peel-1*. We screened ∼30,000 mutagenized genomes, basically as described above, but found only one surviving strain that could be propagated without the multicopy rescuing array. However, follow-up PCR and RNAi experiments showed that the reason for this strain’s survival was the chance integration into the genome of one or more copies of the rescuing construct derived from the original extrachromosomal array.

Overall, even though we did not find mutations that allow ELT-3 to specify endoderm, we conclude that this type of screen is robust, with an acceptably low level of false positives or bypass mutations. Integration into the genome of copies of the rescuing construct occurs at an acceptably low frequency and moreover, is easily detected by PCR.

### Future improvements

We have developed a general strategy to isolate mutations that rescue lethality of *C. elegans* mutants. *tasp-1* and *pqn-82* genes were identified by virtue of multiple alleles detected by whole genome sequencing without the need of prior out-crossing, mapping or complementation testing. Presumably, the remaining strains carry modifier mutations in genes that were hit only once in our screen; such modifiers therefore occur at the rate of ∼1/14000 mutations/gene/gamete and are likely to include (rare) gain-of-function alleles or hypomorphs of essential genes. It is possible that such rare alleles could be detected by increasing the scale of the screen, to the point where these genes would also turn up as multiple independent hits. However, it is important to have an alternative strategy such as out-crossing followed by SNP mapping (Davis *et al.* 2005) or genomic sequencing (Joseph *et al.* 2017). In the present case, we found this was not practical. Outcrossing involved the simultaneous maintenance of the unlinked *caSi2* II MosSCI insertion and the *end-1end-3* V loci, as well as the candidate mutation, with the added complication that the candidate modifier could be acting maternally. Moreover, males from the candidate strains were either difficult to produce or, when produced, did not mate. Thus, any future repetitions of the screen would be best done with a new parental strain in which the *end-1p*::*elt-2* cDNA single copy insertion is linked tightly to the *end-1end-3* locus. We suspect that future screens could be made even more powerful by using a wildtype *end-1* or *end-3* gene as the rescuing extrachromosomal transgene (rather than *end-1p*::*elt-2* with its relatively inefficient rescue), coupled to a lower concentration or a tighter transcriptional control of the heatshock::*peel-1* counterselection plasmid. These straightforward modifications would lead to a healthier parental strain, higher brood sizes following mutagenesis and hence greater numbers of mutagenized genomes that could be easily screened.

### Summary and Conclusions

The screen described in this paper has allowed us to search for modifiers of the ability of a single copy *end-1p*::*elt-2* cDNA transgene to rescue the lethality of the *end-1end-3* double mutant. Through whole genome sequencing, two modifier genes, *tasp-1* and *pqn-82*, were identified and both were validated as loss-of-function modifiers by RNAi and by *de novo* introduction of knockout mutations using CRISPR/Cas9. More work will be required to understand how these two modifiers function at the molecular level but they both seem the type of genes that could influence the shape of a Waddington developmental landscape as depicted on Figure 1. Our plan for the future will be to repeat this screen (with optimizations) on a much larger scale, to better define the transcriptional landscape of the *C. elegans* endoderm and the reasons for its developmental robustness.

Finally, we suggest that the basic design of the present screen—rescuing the lethality of a hypomorph in a parent strain by an extrachromosomal array that can be counterselected following mutagenesis—could easily be applied to other interesting situations in *C. elegans* (see also (Joseph *et al.* 2017)). Such screens could well be simpler and more efficient than more customary approaches using temperature sensitive alleles or classical balancers, or even GFP-marked balancers combined with a worm sorter.
